# Superconductivity in chromium nitrides Pr_3_Cr_10-x_N_11_ with strong electron correlations

**DOI:** 10.1093/nsr/nwz129

**Published:** 2019-09-02

**Authors:** Wei Wu, Kai Liu, Yanjie Li, Zhenhai Yu, Desheng Wu, Yuting Shao, Shihang Na, Gang Li, Ruizhen Huang, Tao Xiang, Jianlin Luo

**Affiliations:** 1 Beijing National Laboratory for Condensed Matter Physics and Institute of Physics, Chinese Academy of Sciences, Beijing 100190, China; 2 School of Physical Sciences, University of Chinese Academy of Sciences, Beijing 100190, China; 3 Department of Physics and Beijing Key Laboratory of Opto-electronic Functional Materials & Micro-nano Devices, Renmin University of China, Beijing 100872, China; 4 School of Physical Science and Technology, ShanghaiTech University, Shanghai 201210, China; 5 Songshan Lake Materials Laboratory, Dongguan 523808, China; 6 Kavli Institute for Theoretical Sciences, Beijing 100190, China

**Keywords:** Cr-based superconductors, nitrides, strong electron correlations, unconventional superconductivity

## Abstract

Exploration of superconductivity in Cr-based compounds has attracted considerable interest because only a few Cr-based superconductors (CrAs, A_2_Cr_3_As_3_ and ACr_3_As_3_ (A = K, Rb, Cs, Na)) have been discovered so far and they show an unconventional pairing mechanism. We report the discovery of bulk superconductivity at 5.25 K in chromium nitride in Pr_3_Cr_10-x_N_11_ with a cubic lattice structure. A relatively large upper critical field of *H*_c2_(0) ∼ 12.6 T is determined, which is larger than the estimated Pauli-paramagnetic pair-breaking magnetic field. The material has a large electronic specific-heat coefficient of 170 mJ K^−2^ mol^−1^—about 10 times larger than that estimated by the electronic structure calculation, which suggests that correlations between 3d electrons are very strong in Pr_3_Cr_10-x_N_11_, and thus quantum fluctuations might be involved. Electronic structure calculations show that the density of states at the Fermi energy are contributed predominantly by Cr 3d electrons, implying that the superconductivity results mainly from the condensation of Cr 3d electrons. Pr_3_Cr_10-x_N_11_ represents a rare example of possible unconventional superconductivity emerging in a 3D system with strong electron correlations. Nevertheless, clarification of the specific pairing symmetry needs more investigation.

## INTRODUCTION

The 3d transition-metal oxides or pnictides exhibit rich quantum phases with novel quantum states, such as long-range magnetic orders, charge or spin density waves, metal-insulator transitions, high-*T*_c_ superconductivity and colossal magnetoresistance. In particular, unconventional high-*T*_c_ superconductivity has been discovered in cuprates as well as in iron-based superconductors [1,2]. Many compounds with 3d transition-metal elements can become superconducting at low temperatures. However, it is relatively difficult to find a superconducting material in chromium-based compounds because most Cr-based compounds have strong magnetism, which generally is not in favor of superconductivity. In fact, CrAs, A_2_Cr_3_As_3_ and ACr_3_As_3_ (A = Na, K, Rb, Cs) are the only Cr-based superconductors so far [3–6].

Superconductivity in CrAs was discovered in 2014 [3,4]. CrAs undergoes a first-order antiferromagnetic transition with a double helical spin structure at *T*_N_ ≈ 265 K [3,7,8]. A bulk superconductivity with *T*_c_ ≈ 2 K emerges above a pressure *P*_c_ ≈ 8 kbar, where the antiferromagnetic order is completely suppressed. Both the NMR and neutron-scattering measurements for CrAs under high pressure revealed that there are strong magnetic fluctuations and line nodes may exist in the superconducting gap function [9–13], as in Sr_2_RuO_4_ or some heavy fermion superconductors [14,15]. After the discovery of superconductivity in CrAs, superconductivity has also been found in the quasi-1D compounds A_2_Cr_3_As_3_ (A = K, Rb, Cs) [3,4]. The upper critical field *H*_c2_ of K_2_Cr_3_As_3_ is about three times larger than the Pauli-paramagnetic limit.

**Figure 1.**
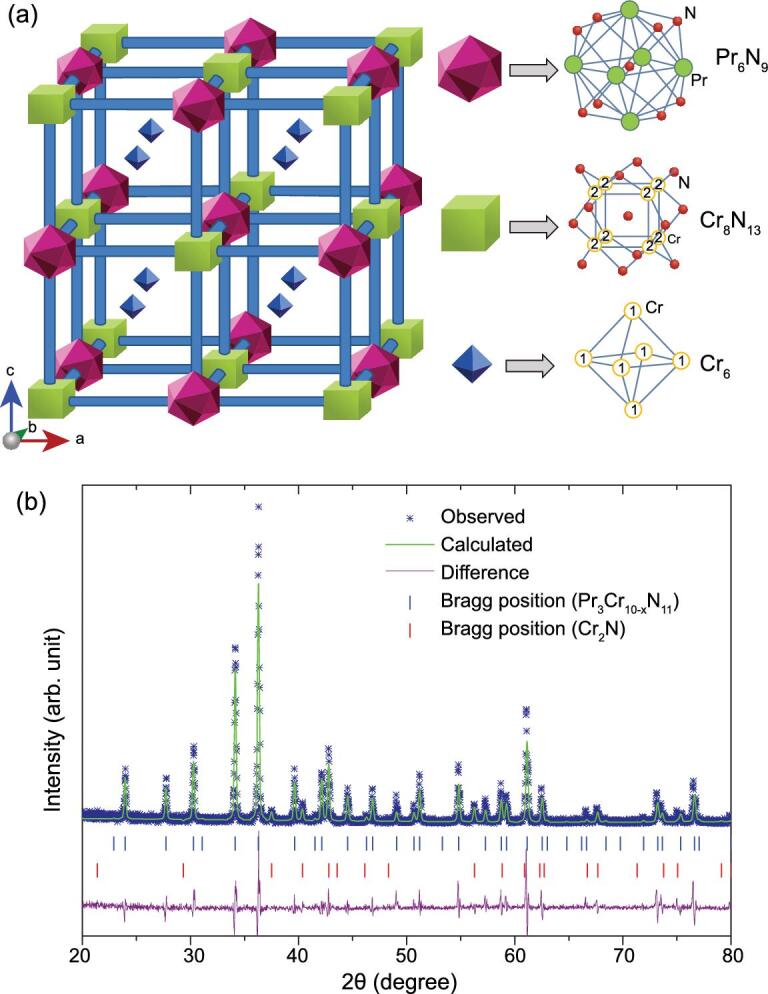
(a) The face-centered cubic cell of Pr_3_Cr_10-x_N_11_ with three kinds of building blocks: Pr_6_N_9_, Cr_8_N_13_ and Cr_6_, respectively. The blocks are shown in a shrunken form. There are two Cr positions: Cr1 and Cr2. (b) Typical Rietveld refinement of Pr_3_Cr_10-x_N_11_ under ambient conditions. The vertical bars represent the calculated Bragg reflection positions of the diffraction peaks for Pr_3_Cr_10-x_N_11_. The difference between the observed (scatters) and the fitted patterns (line) is shown at the bottom of the diffraction peaks.

In this paper, we report a novel Cr-based nitride superconductor with a cubic structure, Pr_3_Cr_10-x_N_11_, which was first synthesized and characterized by Broil *et al.* in 1995 [16]. The compound crystallizes in space group *Fm*-3*m* (No. 225) with lattice constant *a* = 12.891 Å. It contains 192 atoms in a face-centered cubic (FCC) cell with three kinds of building blocks, as illustrated in Fig. 1a. The building blocks are Pr_6_N_9_, Cr_8_N_13_ and Cr_6_. Previous studies showed that there are Cr vacancies in the lattice structure. However, no low-temperature physical properties were reported [16,17]. Our key finding of this study is the observation of superconductivity in Pr_3_Cr_10-x_N_11_ with *T*_c_ ∼ 5.25 K. The high-quality samples have a shielding fraction of 85% at 2 K from zero-field-cooled (ZFC) magnetic susceptibility and a prominent superconducting peak in the specific-heat measurement. A relatively large upper critical field is found at the zero-temperature limit, *H*_c2_(0) ∼ 12.6 T, which is larger than the Pauli-paramagnetic pair-breaking field. From electronic structure calculations, we find that the density of states (DOS) at the Fermi energy are predominately contributed by Cr 3d electrons. The present results demonstrate that Pr_3_Cr_10-x_N_11_ is the first Cr-based superconductor discovered in chromium nitrides and it represents a rare example that possibly unconventional superconductivity emerges in a 3D system with strong electron correlations.

## RESULTS AND DISCUSSION

### Sample and characterization

Polycrystalline samples of Pr_3_Cr_10-x_N_11_ were prepared by direct reactions of the corresponding binary nitrides with solid reaction. Synthesized powder shows a dark-brown color and is air-sensitive, as it is easily oxidized to Pr_2_O_3_ within a few hours. Figure 1b shows the General Structure Analysis System (GSAS) refinement of Pr_3_Cr_10-x_N_11_ under ambient conditions, which indicates that Pr_3_Cr_10-x_N_11_ crystallized in an FCC structure with space group *Fm*-3*m*. All the Pr_3_Cr_10-x_N_11_ reflections can be well indexed based on a cubic cell with lattice parameter *a* = 12.8521 Å, which is consistent with those reported in the literature (*a* = 12.891 Å) [16], indicating their ideal composition. A small amount of raw reactant Cr_2_N was marked with a red vertical bar.

### Temperature-dependent resistivity in Pr_3_Cr_10-x_N_11_

**Figure 2.**
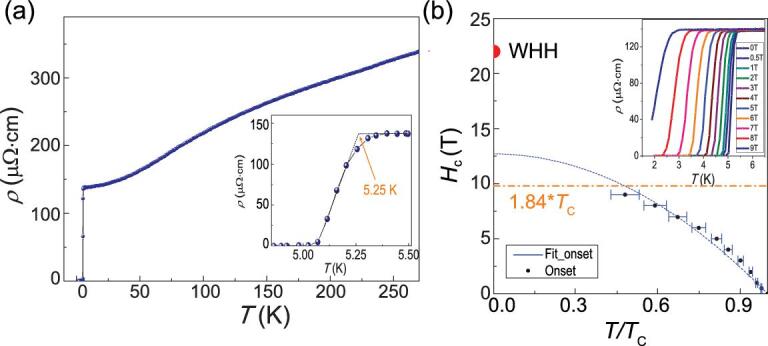
(a) Temperature dependence of the resistivity for Pr_3_Cr_10-x_N_11_ from 1.8 to 300 K at the zero field. The inset shows the resistivity near superconducting transition. (b) The temperature dependence of the upper critical magnetic field. The solid circle shows the upper critical field obtained from WHH fitting. The inset shows the temperature dependence of the resistivity at different fields.

Figure 2a shows the temperature-dependent resistivity for Pr_3_Cr_10-x_N_11_ from 1.8 to 300 K at zero field. The normal-state resistivity is metallic, with no phase transition observed. At low temperatures, a sharp superconducting transition is observed with onset *T*_c_ of about 5.25 K, as shown in the inset of Fig. 2a. The *T*_c_ of Pr_3_Cr_10-x_N_11_ is higher than that of CrAs with *T*_max_ ∼ 2 K under pressure and is close to K_2_Cr_3_As_3_ with *T*_c_ ∼ 6.1 K [12].

**Figure 3.**
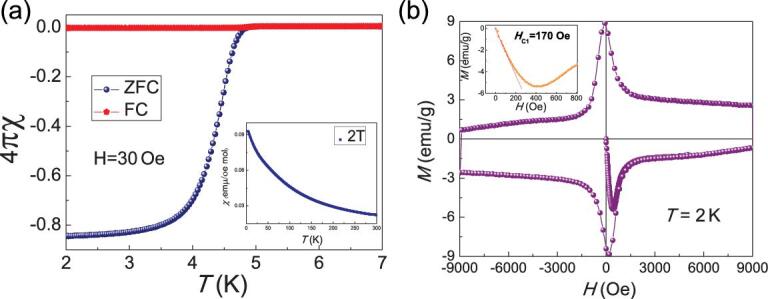
(a) Temperature dependence of the dc magnetic susceptibility with zero field cool (ZFC) and field cool (FC) measured in a field of 30 Oe. The inset of Fig. 3a is the normal-state susceptibility measured in a field of 2 T. (b) The magnetic hysteresis of the sample measured at 2 K. The left inset shows the lower critical magnetic field.

The new superconductor Pr_3_Cr_10-x_N_11_ shows a relatively large upper critical field. Figure 2b shows resistivity data in magnetic fields up to 9 T. As the field increases, the transition temperature *T*_c_ shifts to a lower temperature and the transition width is gradually broadened, similar to the iron-based superconductors [18,19]. The upper critical field *H*_c2_ (T) curve obtained from the field-dependent transition temperatures shows a remarkably high critical field of 12.6 T using the formula *H*_c2_(T) = *H*_c2_(0)(1 − *t*^2^), where *t* is the reduced temperature *t = T/T*_c_ and of 22 T using the Werthamer−Helfand−Hohenberg (WHH) theory [20]. On the other hand, the Pauli-paramagnetic limit for the upper critical field is *H*_P_ = 1.84*T*_c_ ≈ 9.6 T in the case of an isotropic full superconducting gap without considering spin–orbit coupling [21,22]. The *H*_c2_ (0) in Pr_3_Cr_10-x_N_11_ is 130% as large as H_P_. Usually, the high superconducting upper critical field can be originated from multi-band effects, the strong-coupling effect, the spin-triplet pairing and the strong spin–orbit coupling effect in a low-dimensional system [23–25]. The origin of large *H*_c2_(0) in Pr_3_Cr_10-x_N_11_ needs to be further studied. The obtained ${\mu}_0{H}_{c2}(0)$ allows us to estimate the Ginzburg–Landau coherence length $\xi$= 51 Å according to the relationship: ${\mu}_0{H}_{c2}(0)={\Phi}_0/2\pi {\xi}^2$, where ${\Phi}_0$ =2.067 × 10^−15^ Wb is the magnetic flux quantum.

### Magnetic-susceptibility measurements in Pr_3_Cr_10-x_N_11_

The bulk superconductivity in Pr_3_Cr_10-x_N_11_ was confirmed by magnetic-susceptibility measurements. Figure 3a shows susceptibility χ at low temperatures with ZFC and field cool (FC) under a magnetic field of 30 Oe. *χ* starts to drop below *T*_c_ and the diamagnetic signal tends to saturate at low temperatures. The shielding fraction estimated from the ZFC magnetic susceptibility at 2 K is 85%, confirming bulk superconductivity in the sample. The normal-state susceptibility χ increases with decreasing temperature in Pr_3_Cr_10-x_N_11_, showing a Curie–Weiss behavior as shown in the inset of Fig. 3a. Such behavior is different from that of isostructural material La_3_Cr_10-x_N_11_, which shows a Pauli paramagnetism with a nearly temperature-independent susceptibility [16]. Since La^3+^ ion has no occupied 4f electrons while each Pr^3+^ ion has two occupied 4f electrons, it is natural to attribute the Curie–Weiss behavior of χ(T) in Pr_3_Cr_10-x_N_11_ to the magnetic moments of Pr^3+^ 4f electrons. Using a Curie–Weiss fit with formula χ(T) = χ_0_ + C/(T-θ), we obtained the effective moment of each Pr ion at about 3.6 μ_B_, which is very close to the calculated moment of 3.5 μ_B_ for Pr^3+^ by Hund’s rule. The negative value of θ indicates the correlations between Pr ions are antiferromagnetic.

Further confirmation of superconductivity is shown by the magnetic hysteresis of the sample measured at 2 K in Fig. 3b, which displays the typical magnetic hysteresis curve for a type-II superconductor. From the inset of Fig. 3b, the lower critical magnetic fields *H_c1_* of 170 Oe can be obtained.

### Specific-heat measurements

**Figure 4.**
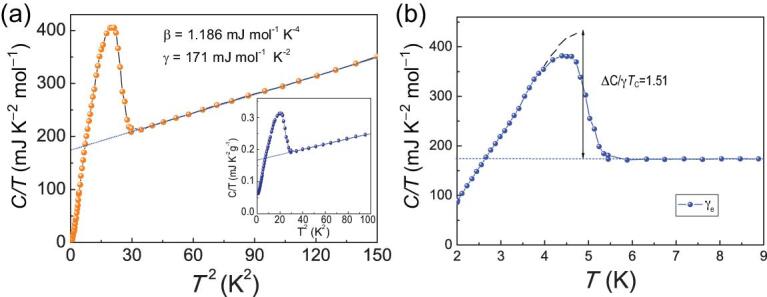
(a) The specific-heat coefficient *C/T* of Pr_3_Cr_10-x_N_11_ as a function of *T* ^2^. The inset shows *C/T* versus *T* ^2^ for a sample with Cr_2_N_._ (b) Temperature dependence of normalized electronic specific-heat *C_e_/* T.

Figure 4a shows the specific-heat coefficient *C/T* as a function of *T*^2^ from 2 to 10 K at the zero field. The bulk nature of superconductivity is confirmed by a pronounced anomaly around *T*_c_ = 5.25 K, consistently with the resistivity and susceptibility measurements. Extrapolating *C/T* to zero temperature gives a residual value of *γ*_0_ = 0.061 mJ g^−1^ K^−2^. As indicated previously, there is raw reactant phase Cr_2_N in the sample. We measured the specific heat of Cr_2_N and found that it can be well fitted by C = γT + βT^3^ below 10 K with γ = 22 mJ mol^−1^ K^2^ and β = 0.0373 mJ mol^−1^ K^4^. Given the residual specific-heat *γ*_0_ origin from the Cr_2_N phase, we can then obtain the specific heat of pure Pr_3_Cr_10-x_N_11_ by subtracting that of Cr_2_N from the total specific heat, as shown in the inset of Fig. 4a.

Above *T*_c_, the good linear *T* ^2^ dependence of *C/T* indicates that the normal-state specific heat consists of two parts of contributions: the electronic part, which is proportional to *T*, and the phonon part, which is proportional to *T*^3^ at low temperatures. By fitting the normal-state specific heat C with the formula *C = γ_n_T + βT^3^*, we find that *γ*_n_ = 173 mJ K^−2^ mol^−1^ and *β* = 1.186 mJ K^−4^ mol^−1^. The normal-state electronic coefficient γ_n_ is proportional to the DOS at the Fermi level. Assuming that most of the DOS comes from the Cr 3d electrons, the value of γ_n_ per mole Cr is *γ*_n_ = 17.3 mJ K^−2^ mol Cr^−1^ for Pr_3_Cr_10-x_N_11_, which is much larger than the corresponding value for CrAs (∼7 mJ K^−2^ mol^−1^) and MnP (∼8.3 mJ K^−2^ mol^−1^) at ambient pressure [11,26] and it is slightly less than that for K_2_Cr_3_As_3_ (∼23.3 mJ K^−2^ mol Cr^−1^) and KCr_3_As_3_(∼27.1 mJ K^−2^ mol Cr^−1^) [14,27,28]. The relatively large *γ*_n_ for Pr_3_Cr_10-x_N_11_ indicates strong correlations of Cr 3d electrons. The Debye temperature *θ*_D_ obtained from *β* is 339 K.

In Fig. 4b, the normalized specific-heat jump at *T*_c_ is found to be Δ*C*/γ_n_*T*_c_ = 1.51. This value is much smaller than those of K_2_Cr_3_As_3_ (∼2.5) and LaNiAsO (∼1.9), which are regarded as strong-coupling superconductivity [14,27,29]. The normalized Δ*C/γ*_n_*T*_c_ reflects the coupling strength between the conducting electrons and the pairing glue. We can then estimate the electron–phonon coupling constant *λ* = 0.6 from the modified McMillian formula [25,30,31]:

$$\begin{equation*}
\lambda =\frac{1.04+{\mu}^{*}\ln (\omega /1.2{T}_c)}{(1-0.62{\mu}^{\ast})\ln (\omega /1.2{T}_c)-1.04}
\end{equation*}$$

where μ^∗^ is a Coulomb pseudopotential and ω is a logarithmic averaged phonon frequency. ω can be determined from the specific-heat jump at *T*_c_ using the formula: Δ*C*/γ_n_*T*_c_ = 1.43[1 + 53(*T*_c_/ω)2ln(ω/3*T*_c_)]. Taking μ^∗^ = 0.10 and *T*_c_ = 5.25 K, we obtained ω = 320 K and λ = 0.6. For such a small electron–phonon coupling constant, the large *H*_c2_(0) value is not likely due to the strong-coupling effect.

### Theoretical calculations

**Figure 5.**
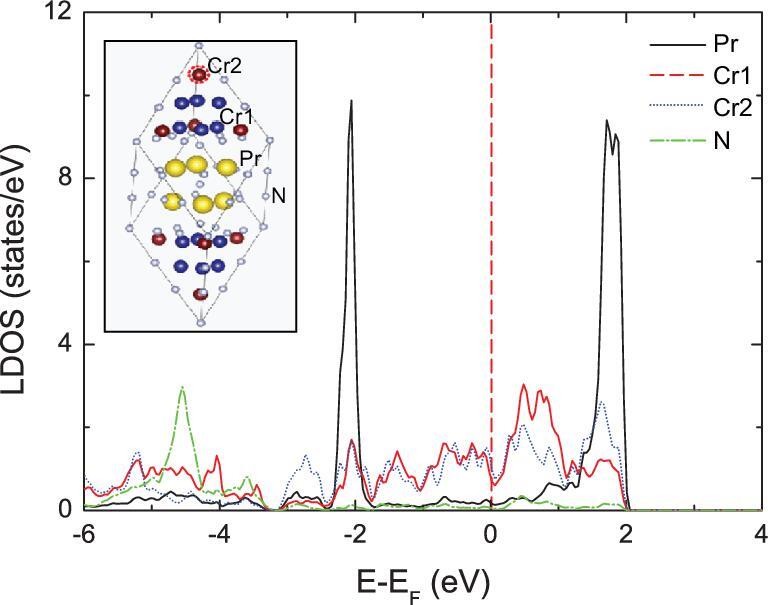
Local density of states (LDOS) for Pr, Cr1, Cr2 and N atoms in Pr_3_Cr_9.5_N_11_. Inset shows a primitive cell with the yellow, blue, maroon and gray balls representing Pr, Cr1, Cr2 and N atoms, respectively. The atomic vacancy at the Cr2 site is highlighted by a red dashed circle.

In order to examine which atomic species contribute most around the Fermi level (E_F_), we have plotted the local density of states (LDOS) for Pr_3_Cr_9.5_N_11_ as shown in Fig. 5. Electronic structure calculations show that the DOS at the Fermi energy are contributed predominantly by Cr 3d electrons, implying that the superconductivity results mainly from the condensation of Cr 3d electrons similar to that in CrAs [32]. The primitive cell of Pr_3_Cr_10-x_N_11_ is shown in the inset of Fig. 5, in which there are two types of nonequivalent Cr atoms, labeled as Cr1 and Cr2, respectively. Both the Cr1 and Cr2 atoms have large contributions around E_F_, mainly originating from the 3*d* orbitals of Cr atoms. In contrast, the vast majority of states for Pr and N atoms are far from the Fermi level. A higher density of Cr2 vacancies, such as in Pr_3_Cr_9_N_11_, does not change the results very much. According to our calculations, the total DOS of Pr_3_Cr_9.5_N_11_ at the Fermi level *N*(E_F_) is 7.37 states/(eV$\ast$f.u.). As a result, the corresponding calculated electronic specific-heat coefficient *γ*_e_ is 17.4 mJ K^−2^ mol^−1^. Experimentally, the measured *γ*_e_ for Pr_3_Cr_10-x_N_11_ is about 173 mJ K^−2^ mol^−1^—about 10 times more than the band calculations, which indicates the strong mass-enhancement effect.

## CONCLUSION

Superconductivity in Pr_3_Cr_10-x_N_11_ shows several novel characters. First, electronic structure calculations show that most of DOS at Fermi energy is contributed by Cr 3*d* electrons, suggesting that the superconductivity is originated from the condensation of Cr 3*d* electrons. So Pr_3_Cr_10-x_N_11_ is the first Cr-based superconductor discovered in nitrides. A few other known Cr-based superconductors (CrAs, A_2_Cr_3_As_3_ and ACr_3_As_3_ (A = K, Rb, Cs, Na)) are arsenide. Superconductivity in CrAs emerges in the vicinity of a quantum critical point and antiferromagnetic spin fluctuations associated with the quantum criticality could act as an important glue medium for Cooper pairing. Superconductivity in the quasi-1D compounds A_2_Cr_3_As_3_ (A = K, Rb, Cs) shows non-s-wave pairing. Both of them show an unconventional pairing mechanism. So the Cr d-electrons play an important role in electron correlations and possibly unconventional superconductivity in Pr_3_Cr_10-x_N_11_ with Cr d-electrons.

Second, Pr_3_Cr_10-x_N_11_ has a relatively large upper critical field *H*_c2_(0) ∼ 12.6 T, exceeding the Pauli limit of the paramagnetic pair-breaking field; this is rare in 3D-structure superconductors. The upper critical field provides very important information on the superconducting pairing. This behavior resembles that unconventional superconducting in K_2_Cr_3_As_3_ in which *H_c2_* is three times the Pauli-paramagnetic limit that is regarded as evidence of spin-triplet superconductivity.

Last but not least, the measured *γ*_e_ for Pr_3_Cr_10-x_N_11_ is about 173 mJ mol^−1^ K^−2^, equivalent to 17.3 mJ K^−2^ mol Cr^−1^. This *γ* value is close to that of K_2_Cr_3_As_3_ (mJ K^−2^ mol Cr^−1^), indicating enhanced electron correlations in Pr_3_Cr_10-x_N_11._ The experimental value of *γ*_e_ is about 10 times higher than the electronic calculations. This large renormalization factor cannot be explained by electron–phonon interactions and thus quantum fluctuations might be involved.

In conclusion, we report the experimental result for a novel Cr-based superconductor nitrides Pr_3_Cr_10-x_N_11_ with a cubic lattice structure. Bulk superconductivity with *T*_c_ ∼ 5.25 K is observed from the resistivity, susceptibility and specific-heat measurements. Further theoretical and experimental studies are needed to determine the pairing symmetry and the corresponding mechanism, especially the role of Cr 3d electrons, for the observed superconductivity in Pr_3_Cr_10-x_N_11_.

## METHODS

### Sample preparation

Polycrystalline samples of Pr_3_Cr_10-x_N_11_ were prepared by direct reactions of the corresponding binary nitrides, staring with PrN (99%) and a mixture of the chromium nitrides (CrN and Cr_2_N) in a mass ratio of 4:6. The operations were all performed in an Ar-filled glovebox. Cold-pressed pellets of the mixtures were sealed in an evacuated quartz tube (<10^−4^ Pa). The pellets were gradually heated in 1 day to 1000°C, held at that temperature for 50 hours, then cooled to the room temperature in the furnace. The products were again ground in a glovebox, pressed into pellets, wrapped in Ta foil and heated in an evacuated quartz tube at 1165°C for 120 hours. It is worth noting that, after this treatment, the samples were usually still contaminated by the binary nitrides. The treatment of these samples with diluted hydrochloric acid only dissolved the rare-earth nitrides. In between these treatments, the pellets were ground to a fine powder, the decomposed rare-earth nitride was dissolved in hydrochloric acid and fresh rare -earth nitride was added again to the mixture to maintain the proper composition. The reaction temperature was carefully selected to avoid decomposition of the products, meanwhile obtaining a good crystallization. In our study, we found that Pr_3_Cr_10-x_N_11_ partially decomposed above 1200°C. Synthesized powder shows a dark-brown color and is air-sensitive, as it is easily oxidized to Pr_2_O_3_ within a few hours.

### Measurements

The electrical transport measurement was carried out on a physical-property measurement system (PPMS-9, Quantum Design). The resistivity was measured by a standard four-probe method, employing silver-paste contacts cured at room temperature, used for resistivity measurements, with the electric current applied in an arbitrary direction. The magnetic susceptibility was measured in a Quantum Design SQUID VSM. The specific-heat measurements were performed up to 9 T in a PPMS.

### Theoretical modeling

We carried out first-principles electronic structure calculations on Pr_3_Cr_10-x_N_11_. The first-principles calculations were performed using the projector augmented-wave method [33], as implemented in the VASP package [34]. The generalized gradient approximation of the Perdew–Burke–Ernzerhof type [35] was adopted for the exchange-correlation function.

## Supplementary Material

nwz129_Supplemental_File
