# Supplementary material for: Superconductivity in chromium nitrides Pr3Cr10-xN11 with strong electron correlations
Source: Natl Sci Rev. 2019 Sep 2;7(1):21–6. doi: 10.1093/nsr/nwz129 (PMC8288919; doi:10.1093/nsr/nwz129)
Supplement: nwz129_Supplemental_File [file nwz129_supplemental_file.docx]

**Supporting Information**

**Superconductivity in** **Chromium Nitrides Pr_3_Cr_10-x_N_11_** **with Strong Electron Correlations**

Wei Wu^1, 2^, Kai Liu^3^, Yan Jie Li^1, 2^, Zhen Hai Yu^4^, De Sheng Wu^1, 2^, Yu Ting Shao^1, 2^, Shi Hang Na^1, 2^, Gang Li^1,5^, Rui Zhen Huang^2,6^, Tao Xiang^1,2,6^& Jian Lin Luo^*1, 2, 5^

*^1^Beijing National Laboratory for Condensed Matter Physics and Institute of Physics, Chinese Academy of Sciences, Beijing 100190, China*

*^2^School of Physical Sciences, University of Chinese Academy of Sciences, Beijing 100190, China*

*^3^ Department of Physics and Beijing Key Laboratory of Opto-electronic Functional Materials & Micro-nano Devices, Renmin University of China, Beijing 100872, China*

*^4^School of Physical Science and Technology, ShanghaiTech University, Shanghai 201210, China*

^5^*Songshan Lake Materials Laboratory, Dongguan, Guangdong 523808, China*

*^6^Kavli Institute for Theoretical Sciences, Beijing 100190, China*

*Correspondence should be addressed to J. L. Luo ( [*jlluo@iphy.ac.cn*](mailto:jlluo@iphy.ac.cn) )

Contents

**Experiments and Calculations**

**Figure S1 ............................................................................................................................... S2**

**Figure S2 ............................................................................................................................... S2**

**Figure S3 ............................................................................................................................... S4**

**References ............................................................................................................................. S5**

**Experiments：**

The crystal lattice parameters of the samples play an important role in the observation of superconductivity. As shown in Figure S1, if we compare three samples with different *T*_c_, we noticed that the main x-ray diffraction peaks of [333] of these samples are also different. The *T*_c_ increases from 3.7 K to 5.25 K while the angle for peak [333] position decreases, and the transition is getting sharper.


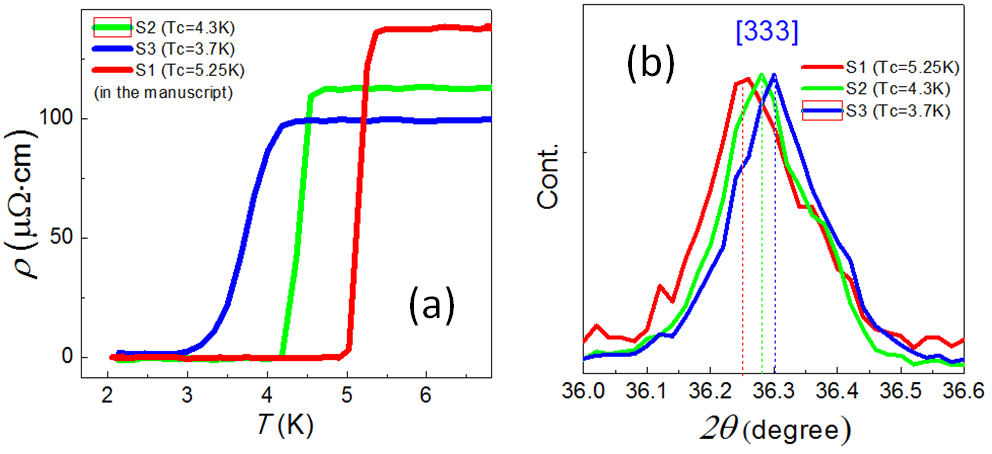


**Figure S1** (a) Temperature dependence of the resistivity for Pr_3_Cr_10-x_N_11_ with three different *T*_c_ samples. (b) The main X-ray diffraction peak of [333] about Pr_3_Cr_10-x_N_11_ with three different T_c_ samples

***
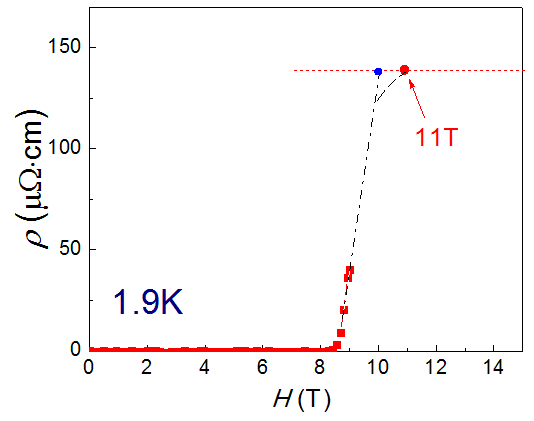
***

**Figure S2** (a) Magneto-resistivity at 1.9K in sample 1(*T*_c_=5.25K). The red dotted line locate at the value of normal state resistivity above Tc.

***Calculations:*** We have carried out first-principles electronic structure calculations on Pr_3_Cr_10-x_N_11_. The first-principles calculations were performed by using the projector augmented wave (PAW) method**^1^** as implemented in the VASP package.**^2^** The generalized gradient approximation (GGA) of Perdew-Burke-Ernzerhof (PBE) type was adopted for the exchange-correlation functional.**^3^**

The orbitals of Pr (5s^2^5p^6^4f^2^6s^2^5d^1^), Cr (3p^6^3d^5^4s^1^), and N (2s^2^2p^3^) were treated as valence electrons. The kinetic energy cutoff of the plane-wave basis was set to be 520 eV. A 6×6×6 *k*-point mesh was employed for the Brillouin zone sampling of the primitive cell of Pr_3_Cr_10-x_N_11_, which contains two formula units (f.u.) [Fig. S3(b)]. The Gaussian smearing method with a width of 0.05 eV was used for the broadening of Fermi surface. The on-site Coulomb repulsion among the localized *d* or *f* electrons was included by using the GGA+U formalism of Dudarev *et al.***^4^** The values of effective Hubbard U for Pr 4f and Cr 3d electrons were set to 5.0 eV and 3.0 eV, respectively. The lattice constant of Pr_3_Cr_10-x_N_11_ was fixed at the experimental value of 12.891(2) Å. The Cr vacancy in Pr_3_Cr_10-x_N_11_ was studied by subtracting real Cr atom from the primitive cell. The internal atomic positions were allowed to relax until the forces on all atoms were smaller than 0.01 eV/Å.

The primitive cell of Pr_3_Cr_10-x_N_11_ is shown in Fig. S3(b), in which there are two types of nonequivalent Cr atoms, labeled as Cr1 and Cr2, respectively. By comparing the cohesive energies of the Pr, Cr1, Cr2, and N atoms in Pr_3_Cr_10-x_N_11_, we learn that the Cr2 atom has the lowest cohesive energy (0.83 eV), which is in accordance with the experimental findings that the Cr2 site is most prone to form vacancy. The defective Pr_3_Cr_10-x_N_11_ has then been simulated by subtracting one Cr2 atom (highlighted by a red dashed circle) from the primitive cell, which corresponds to a chemical component of Pr_3_Cr_9.5_N_11_. The band structure of Pr_3_Cr_9.5_N_11_ along the high-symmetry paths of Brillouin zone is shown in Fig. S3(a). Obviously, there is one hole-type band crossing the Fermi level around the X point, being consistent with the cation deficiency in Pr_3_Cr_9.5_N_11_.


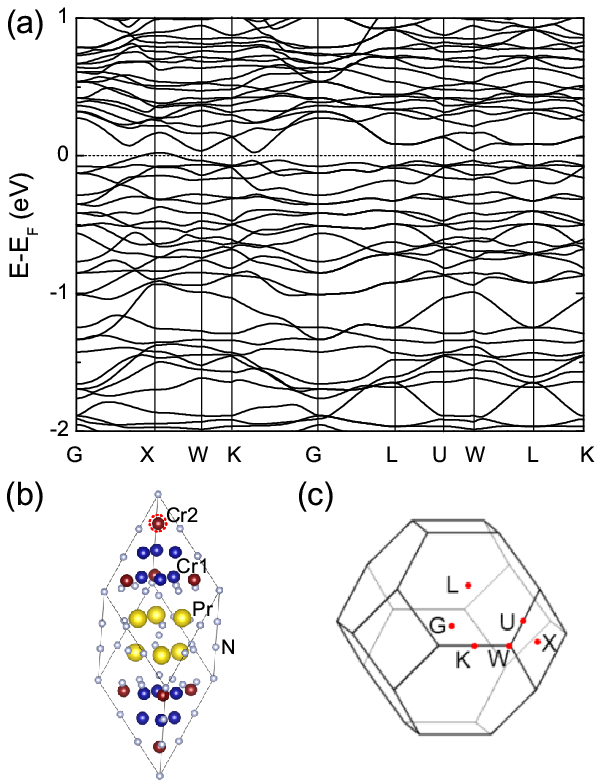


**Figure S3** (a) Band structure of Pr_3_Cr_10-x_N_11_ along the high-symmetry paths of Brillouin zone. (b) Primitive cell and (c) Brillouin zone of Pr_3_Cr_9.5_N_11_. The yellow, blue, maroon, and gray balls represent the Pr, Cr1, Cr2, and N atoms, respectively. The atomic vacancy at the Cr2 site is highlighted by a red dashed circle.

**References:**

(1) Blöchl P E. Projector augmented-wave method. *Phys Rev B* 1994; 50:17953-17979.

Kresse G and Joubert D. From ultrasoft pseudopotentials to the projector augmented-wave method. *Phys Rev B* 1999; 59:1758-1775.

(2) Kresse G and Hafner J. *Ab initio* molecular dynamics for liquid metals. *Phys Rev B* 1993; 47:558-561.

Kresse G and Furthmüller J. Efficiency of ab-initio total energy calculations for metals and semiconductors using a plane-wave basis set. *Comp Mater Sci* 1996; 6:15-50.

Kresse G. Efficient iterative schemes for ab initio total-energy calculations using a plane-wave basis set. *Phys Rev B* 1996; 54:11169-11186.

(3) Perdew J P, Burke K and Ernzerhof M. Generalized Gradient Approximation Made Simple. *Phys Rev Lett* 1996; 77:3865-3868.

(4) Dudarev S L, Botton G A, Savrasov S Y, et al. Electron-energy-loss spectra and the structural stability of nickel oxide: An LSDA+U study. *Phys Rev B* 1998; 57:1505-1509.
